# The Pandemic in India and Its Impact on Footloose Labour

**DOI:** 10.1007/s41027-020-00285-8

**Published:** 2020-10-30

**Authors:** Jan Breman

**Affiliations:** University of Amsterdam, Amsterdam, The Netherlands

**Keywords:** Pandemic, Lockdown, Footloose labour, Circular migration, Self-reliance, Pauperism

## Abstract

The COVID-19 pandemic is deepening the divide between people on the safe side of the social order and those at risk. The former are not only better equipped to protect their immunity, but can also count on support and care if they become infected. In a civilization haunted by the purity–pollution syndrome, the virus amplifies the stigma of impurity in which substantial segments of the population are forced to work and live. Social distancing fits well with a customary code of segregation. The transition to an informalized economy should be seen in the context of India’s ingrained social inequality resulting in widespread pauperism. In the havoc the pandemic created, politics and governance have further distorted the already highly skewed balance between capital and labour. An overview of the impact of the pandemic on the workforce kept adrift should also allow for the regional diversity that exists. Underlying my appraisal is the anthropological research I have carried out in the state of Gujarat, a major destination for footloose labour from other parts of the country. Since circular migrants are not allowed to settle down and set up home where they have gone to, they are bound to return to their place of origin after shorter or longer bouts of casual employment without effective legal protection and social security. They are kept floating because both capital and government want it that way.

## Deprived of Livelihood

In India, the pandemic is sweeping through a landscape of labour in which the crisis of survival was already chronic in the countryside, but increasingly also in the slums of the rapidly growing mega-cities. Dispossessed from agrarian property sufficient for viable livelihood, the land-poor and landless classes, in particular, are in frantic search of employment outside of agriculture, which from generations used to be the prime sector of the economy. Agricultural production has declined to less than 15% of national income, but is still reckoned to employ about two-fifth of the economically active population. Market liberalization has brought robust growth, but, by replacing labour with capital, it has not created the multimillions of jobs to satisfy the searing need for them.

The lockdown announced on 24 March 2020 gave people 4  hour to prepare for housebound life. In his televised address, the Indian Prime Minister, Narendra Modi referred to the Hindu epic Ramayana, warning his audience, fearful of the virus, not to move out for the next 21 days since the ‘*Lakshman rekha* is at your doorstep’. The vast majority of the footloose proletariat, compelled to roam around in search of gainful work not available locally, was rendered unable to return to their faraway homes.

What is the magnitude of this army of labour leaving their place of residence in the countryside where they are surplus to demand, but firmly prevented from settling down elsewhere? Counts vary from at least 50 million to more than double that number. Footloose workers and their erratic mode of employment defy formal registration, but, in my guesstimate, it accounts for at least one-fourth of the rural workforce and thus constitutes a major segment of the country’s economically active population estimated to be between 450 and 475 million. Migrant workers are torn loose from their rural place of origin for variable periods of time ranging from a couple of weeks, a season or a few years to working life duration. This category does not include people of working age who have managed within the last half century to leave and settle down more definitely wherever they have gone. These migrants who do not come back to the village other than for short visits enjoy higher and steadier incomes enabling them to establish a permanent foothold of sorts and often bringing along dependent members of their household to the home set up in the place of arrival. Usually originating from castes–classes higher up in the village hierarchy, they come already better equipped with physical and social capital. This differential outcome shows that labour migration should be analysed as an elastic and composite concept. Classifying people as either footloose or settled suggests a fixity that obscures a more complex fluidity between mobility and domicile (Srivastava 2020). Addressing them as seasonal labour is problematic as the timeline is not clear and it can vary between short sorties for a couple of weeks to an uneven rhythm of coming and going off again until the end of working life, when these people, worn out already from the age of 40 onwards, are no longer deemed fit for further employment.

The distinction often made between the wet and dry season, however, makes sense because wage work carried out in the open air comes to standstill with the outbreak of the monsoon. But the labour process in factories and workshops has a different kind of seasonality. In my fieldwork stints in Gujarat, I found that labour employed, for example, in dyeing and printing mills and power-loom workshops tried to drop out in the heat of summer to recuperate in the village from their burdensome, stressful, noisy, ill-ventilated and hazardous worksites.

Instead of seasonal labour migration, circular migration would be the better term for the workforce perpetually though erratically kept adrift. The sliding scale between temporary and permanent movement, coincidental with the unsettled versus the settled switch in the site of work and residence, can only be ex post facto determined. Already for more than two to three generations, India’s politics and policies have insisted that a sizable part of the labouring poor leave their rural abodes and the comfort of their family to earn a meagre livelihood for their household in rural or urban locations far away from home. This is a modern form of nomadism to ensure that the workforce at the bottom of the economy, shorn off social security and protection, can be bought at the lowest possible price and only hired for as long as their services are required.

The day after the lockdown order was issued, economic life came to a standstill. An estimated 130 million people lost their jobs, irrespective of how short and precarious these may have been, often even without being paid. A survey by ActionAid towards the end of May 2020 reports that over three-fourths of respondents said that they had lost their livelihood since the imposition of the lockdown. Close to half of the respondents shared that they had not received any wages, while about 17% had received only partial wages. Approximately 53% responded that they had incurred additional debt during the lockdown. More than half of the respondents who had migrated for work were stranded for over a month (ActionAid 2020). A significant detail to add here is that women workers, already highly vulnerable in a saturated labour market, were worse hit than their male counterparts. Women are overrepresented in sectors such as domestic work, construction work, beauty and wellness industry, and sex work, which have seen massive losses of livelihood since the lockdown. Even in the formal sector, women are more likely to be hired on temporary or part-time positions, making it easier for firms to let them go if there is downsizing (ActionAid 2020). Also, the self-employed in the bottom tiers of the economy lost whatever they could do in lieu of a proper job.

With no income to spend on their daily needs—food and cost of makeshift or rental accommodation—and fear from the mushrooming virus, the footloose among them, in particular, were eager to return to their places of origin. However, the ban imposed on all modes of transport and any kind of movement prevented them from returning to their native place. Fear of hunger, lack of shelter and fright of becoming infected, left many among them with no other option than to try and bridge the distance on foot. In the first few days after the instructions were issued to stay indoors, the newspapers were filled with pictures of groups of migrants seen walking and hitchhiking along highways in the hope of completing the journey of hundreds of kilometres or more to their homes. At state and district borders, they were stopped and often harassed by the police who refused passage and interred their catch in temporary camps. Most migrants wisely decided to stay put until long-distance transport would resume. When that did not happen, they gathered in hundreds and thousands at railway stations and bus depots or assembled outdoors in street protests. For instance, they gathered thrice between April and June in Surat city clamouring for vehicles or trains to fetch them back to their places of residence. The police responded to this growing unrest heavy-handedly. But the images of this restive and seething mass at least made the low-key presence of these footloose labourers visible to the urban citizenship, hitherto unaware of the sheer size and vulnerability of this alien workforce in their midst.

## The Identity of Footloose Labour

Who are these people forced to remain circulating between their place of residence and worksites, far away from home? What they have in common is the need to cope with the chronic indigence of the household to which they belong. Shorn of means of production sufficient to live on, there is also no regular and steady demand for their labour power in or around the rural locality inhabited by them. Stuck close to or at the bottom of the informal economy, which implies the on-and-off call on their availability, they have no say whatsoever over the terms of employment. Many of them tend to be hired and fired according to the need of the moment, are paid cheaply for their low-skilled drudgery and have to work without minimal access to legal protection or social security. Migrant workers can be found across all the sectors of the economy, contracted and subcontracted, as casual hands, or in self-employed micro-ventures. Based on their primary identities, they occupy different niches in the labour market. While facilitating the entry of newcomers from their own caste, tribe or creed, they try to secure such conquered sites of employment from intrusion by outsiders with whom they share no bonds. Class-wise, they can be clubbed as either semi-proletarians equipped with meagre and low yielding means of production (land, tools, cattle) or proletarians who are fully dispossessed from such ownership and at risk of even having forfeited control over where and when to apply their labour power. Their social profiles are structured on the basis of their primary identities defined by caste (Scheduled Castes or Dalits, Other Backward Classes), tribe (Scheduled Tribes or Adivasis) or creed (Hindus or Muslims). All these distinct clusters are further subdivided into a broad and stratified repertoire of hierarchical differentiation.

The status of the communities in the lower echelons of the social pyramid corresponds with their similar placement in the economic classification. As migrants, all of them face the same predicament, but this huge army of labour held in reserve is split up in heterogeneity. Most of them are men, but a considerable, though heavily underestimated, portion happens to be women and children, frequently put to work as unpaid helpers. Their massive presence is scattered over a very wide spectrum of occupational differentiation and social segmentation. It is a convoluted divide that also pervades the internal ranking of each of these distinct categories hovering over the official poverty line, which has been fixed at an extremely low level. Vulnerable already in their fragmented mode of existence before departure, most of them remain so on arrival in another state. From day one, they are marked as outsiders lacking local language proficiency and familiarity with the alien habitat and its social intercourse. A drift between their place of origin and the work that entices them away, labour nomads are not without assertiveness. However, it is a resilience that does not amount to a joint platform of protest and resistance. This inability to seek each other out in mutual support to overcome their exclusion from regular employment, from decent pay for their toil and from settled livelihood which would allow them to congregate in concerted action, results in their muted public voice and visibility.

Did the country’s top leadership not realize the impact of the order to stay indoors would have on these workers far from home who were robbed overnight of the meagre fruits of their labour? The curfew announced without former intimation was accompanied by the Prime Minister’s call on employers not to lay off their workers, although it could easily be surmised that in the casual hire-and-fire of labour, the appeal and the directive that followed would be made in vain and would remain unimplemented. He also implored the common weal to be kind and generous to the poor around them. The support that the central government made available to alleviate dire needs, under its *Garib Kalyan Yojana*, amounted to less than 1% of GDP. The Prime Minister implicitly coined a rationale to provide such meagre relief a month later saying that the people of India must learn to be *‘atmanirbhar’*—self-reliant. The implied morality seems to be addressed strictly to the labouring poor only. This political dictum is directly at odds with the government’s eagerness to please capitalist business to the hilt, unwaveringly conceding to the maxim of cheap, pliable and unorganized labour. The premeditated instruction to rely on self-employment and self-sufficiency is, of course, not applicable to the better-off classes. The well-settled bourgeoisie is accustomed to being surrounded by an assortment of menials—cooks, cleaners, sweepers, washers, caretakers of minors and seniors, drivers, gardeners, security guards—to enjoy their leisurely lifestyle. Even petty bourgeois households seek the comfort of one or two part-time domestic helps, in many cases coming from other states.

What has been the impact of the lockdown decision in the first instance? An early report from the Stranded Workers Action Network (SWAN) described the predicament of migrants who got stuck when they dared to walk back home, caught somewhere in between the home they were trying to reach and the work site left behind. The great majority were men, mainly from eastern Uttar Pradesh, Bihar, Jharkhand, Odisha and Madhya Pradesh. Almost three-quarters had food supplies for no longer than 2 days, which they tried to stretch out over more days. Of the daily wage earned—an average of 400 rupees (roughly five US dollars). More than two-thirds of them were left with only 200 rupees or less at the time of contact. Except for an almost negligible fraction, they had received no cash allowances from the government. Nine out of every ten had been dismissed by their employers without wage payment. Migrants said to be even more afraid of the hunger awaiting them than of catching the virus. And of course, they all suffered the trauma of being stranded between their places of work and residence with no prospect of release from the deadlock on either side (SWAN 2020a; Jan Sahas 2020; Centre for Equity Studies 2020). A repeat survey by SWAN nearly 2 months later found that two-thirds of the migrants were still stuck at the same place they were in when the lockdown was imposed. Of those who had reached home—usually by bus or train—SWAN (2020b) reported that a very large majority incurred high costs in making the journey, an expense which added to their spiralling indebtedness, although the Supreme Court had asked the state governments to bear the outlay. Four-fifths of all the respondents had also not received the extra food ration that the government had promised to provide and the migrants still away from home had remained unable to access the Public Distribution System (PDS).

The Ministry of Consumer Affairs, Food and Public Distribution admitted that the process of identification of genuine beneficiaries would take more time (Agarwal 2020). What this really meant is that the machinery of food grain distribution remained almost completely ineffective as far as the migrants impacted by the crisis were concerned. A modest relaxation of the lockdown in early May was accompanied by the resumption of interstate public and private transport. But many migrants continued to be stranded, either because their home state did not allow their return without a proper health check to verify they were asymptomatic or because, in any case, they could not obtain a valid, ‘paid for ticket’ to their destination. Moreover, the host states, apparently under pressure from employers’ lobbies, cancelled trains and buses, afraid that the required workforce would not be around when the economy was going to be kick-started back to normalcy (Wire 2020a). Lack of coordination between the states further botched up the belated assurance of cost-free transportation. Many footloose migrants continued to swarm the roads in frantic attempts to reach home. In their tortuous trek, several hundreds of them died from fatigue, starvation, failing health or accidents when passing through habitat unwilling to grant permission to proceed to these unwanted and possibly virus affected flotsam and jetsam.

Migrants who were able to find waged or self-generated employment within their own state at a longer or shorter distance away from domesticity, by and large, managed to return to their homes in the weeks following the lockdown by avoiding main roads and police patrols. It means that in all likelihood about 40% of all labour nomads had managed by early June to join the households they had left behind. But this time they came back penniless, without the usual savings from their wages, with which their families used to keep themselves afloat. Even more dismal was the plight of those who already had left for work in debt, accepting a much-needed loan which they had to pay back with a monthly interest of 5 to 10%—by working it off. Unable to meet the terms of the loan, they also stand to lose the collateral, aggravating their state of dispossession. The return home of the huge reserve army of labour from the host states has haltingly taken place. Official efforts to block their departure were increasingly made in vain. Having been driven back and forth for many years, they are accustomed to the hire-and-fire operation under which their labour power is mobilized. This time, however, the termination of their services came out of the blue and staying on was no option. Not only for lack of income to spend on daily maintenance, but also because their impure and sullied way of life has been seen as a threat to the well-being of the settled classes who need them when at work, but not as co-residents in nearby and touching vicinity. As already observed, their massive presence does not allow them to team up in togetherness and raise their voice as a collectivity.

## Coping with the Lockdown and Collapse of Public Health Care

What kind of safety net has the government provided to alleviate further destitution? The poor who are entitled to monthly food rations at a reduced price under the public distribution system (PDS) can consider themselves fortunate. Migrant workers absent from the place in which they have been registered for the grain subsidy could not claim such allowance provided in kind. Once dismissed from work, they were anxious to return to their places of residence, where some, at least, could have possibly qualified for the distributed ration. The panic flight from the cities was driven by despair and distress trying to get away from imminent disaster caused by the mix of instant loss of income, access to informal social safety nets at home and fear of the pandemic looming ahead. On their eventual return, the migrants faced strong suspicion that they might be carrying the malignant virus. Not infrequently they were compelled to isolate themselves for the duration of the incubation period, even when no shelter was made available to them. Not only villagers from higher castes insisted on their isolation, but also, quite often, neighbours and kinsmen.

The very uneven regional spread of COVID-19 at least partly reflects not so much differences in the real incidence of the disease, but its lack of detection in many states (Wire 2020c). Public health care, never extensively accessible and cost-free, was subject to far-reaching cutbacks from the end of the twentieth century onwards together with several other pro-poor benefits. The labouring poor remain mainly dependent on quacks and self-medication paid out from their own pocket for their failing health. Many of them die for want of food and lack of medical care, but often neither the nature of their fatality nor its occurrence gets formally notified. They pass away as undocumented in their informality, as they were when still alive. While the pandemic seems to be less deadly in relation to the size of the population, than in countries with much better healthcare provisions, it does not mean that the virus will linger on and finally taper off without causing much harm and distress. Also mortality has been undercounted to an extent which is impossible to estimate (Banaji 2020; Pulla 2020).

Proposals have been submitted from various quarters to assess the huge damage that the pandemic has caused to society and economy, the costs involved in combating it effectively and how to find the necessary funds. The government is addressed under the assumption that it will not fail to see the urgent need of these remedies. In my view, this is an erroneous expectation as governance has fallen far short in deciding on the appropriate strategy and mobilizing the resources to implement it.

Many experts on the PDS have argued that the substantial stores of staple food that have been built up ought to make it possible to extend the system to provide universal relief in the coming months. Scaling up the public works programme in rural areas and extending it to towns and cities would also be an obvious panacea. Others suggest introducing a basic monthly cash benefit up to 10,000 Rupees to all those who are victims of the no-work, no-wage rule. More radical is the proposal to fund poor relief by taxing the mega-rich—a measure that would make the pledge of redistributive equality, not a complete joke. All this sound advice seems to assume that the government is serious about honouring its often repeated pledge that everyone is meant to share in the common good and can rely on public relief to deal with setbacks that they cannot survive under their own steam. The dilution of the labour legislations has in recent years been matched by changes ‘to free employers from unnecessary inspection’. In a sequence to this hands-off policy, the lobby of big business seems to have successfully pressurized the government for a three-year suspension of nearly all labour legislation. This proposal has already been readily accepted as appropriate by several state governments wishfully hoping to lure away multinational capital from East Asia with the promise of ultra-cheap labour to which all bargaining power is adroitly withheld.

## The Failing Remedy of ‘Self-reliance’

Protests against the worsening predicament of the labouring poor are routinely put aside. The standard response to such criticism, if acknowledged at all, has been that their pitiable plight is due to the faint to feckless efforts of the victims themselves and not caused by the lack of official will to moderate their suffering. Mid-May, the government announced a more sizable relief scheme which has already been alluded to as the Self-Reliant India Scheme (*Atma Nirbhar Bharat*), less than 10% of which was set aside for the labouring poor to mitigate their economic distress. The targeted beneficiaries were identified as migrant workers, daily wage earners and self-employed. The scheme aimed at providing food security as well as small and highly elusive cash transfers to keep them afloat for 2 months, and it purported to scale up from the negligible support notified in the first round, but remained solidly framed within the main policy objectives of professed ‘self-reliance’, a small government and privatization of transactions, all of which are core tenets of neoliberalism. The emphasis on self-help sits uneasily with the strenuous efforts by the government to attract foreign investment. Comments in the media on the new reform package were sceptical about the veracity of what is on offer. Balakrishnan aptly commented that to rebuild the economy, India needs to be *atmanirbhar* in ideas (Balakrishnan 2020).

The paucity of reliable data on the staggering unemployment and the excessive burden of deprivation is not the consequence of administrative oversight or sloppiness in measurement. It is intentional and designed to distract attention from the dwindling scope for survival in the broad underside of society. The regime of exploitation and repression which shapes India’s political economy is the driving force by which a major segment of the workforce is kept footloose. The contrast between home states (sending migrants) and host states (receiving migrants) is too simple and should not be reified. Gujarat happens to be a state of both in-migration and out-migration and it is not the only one. In earlier publications (Breman 1996, 2013), I have argued that the demand for outside labour is not necessarily caused by a lack of local supply. In the villages of my fieldwork, a large number of migrants have been employed because they are cheaper, as well as by necessity more docile and pliable than the local landless. The latter are abundantly available throughout the year, but remain unemployed and are forced to search for work elsewhere (Breman 2019–2020). In reporting on labour migration, the government omits the collection of data that would highlight the acts of omission and commission of official agencies engaged in covering up what amounts to human trafficking. Beyond my contention that the government is well known to lack basic information on the scale, shape and employment modality of the workforce kept footloose, my even more disquieting conclusion is that India’s ruling class does not want to know the ins and outs of labour migration.

The flight of migrant labour in the onset of the pandemic has nearly exclusively dealt with their exodus from mega-cities, while the impact of the lockdown on the very sizable rural-to-rural circulation for waged work—crop harvesting, road construction, in brick kilns, stone quarries, sand digging in river beds, saltpans and ship-breaking on the shore—finds hardly any or no mention at all in the reporting. A clear example of neglect is also the absence of documentation on the fate of the footloose workers toiling in the Special Economic Zones (SEZs). These areas have been carved within the states in which they are situated and handed over to mercantile–financial capitalists, several of whom have been considered to be cronies of the political regimes. We do not know what is going on in these safe havens of big business since such enclaves are fenced off from outside, with security guards manning the check-posts. What has happened when the lockdown was declared to this migrant workforce? For sure, laid off instantly, but without transport made available, they were unable to bridge the vast distance separating them from home (Kale 2020). In response to a RTI, the office of the Chief Commissioner of Labour in the central government acknowledged inability to provide information on the labour nomads who got stranded on their way back home. His instruction to report their numbers by states and districts failed to be given effect to (The Statesman 2020; Nayak 2020). A few state governments seem to have been more effective in their handling of the fabricated mess. Kerala, for instance, immediately opened camps where migrants, labelled as guest workers, could take refuge and be apparently treated as human beings.

Alarmed by the pathetic sight of people now visibly adrift, various suggestions have come from voluntary associations to cater to the needs of this floating mass deprived of settled life and work. To start with, they should be registered on arrival in order to facilitate their access to the rights of citizenship and be given access to rental housing at an affordable cost. Such proposals have their origin in the idea that labour nomads deserve to be treated as human beings and not as a commodity cyclically put to use or disuse, shifted around in time and space without redeeming them from endless circulation between home and work. Having investigated the ordeal of the hordes, squatting down in Ahmedabad on unoccupied public land in makeshift accommodation or as pavement dwellers, I ended my last stint of fieldwork in the city on this pauperized lot going around the night shelters for the homeless that urban corporations are since 2009 legally bound to provide, one for every 100,000 inhabitants. To the extent that they have come up, far below the prescribed number, I found these transitional housing facilities managed in public–private partnership (PPP) either in a sorrowful state or not functional at all (Breman 2015). Beginning to address the gap between the shameful absence of adequate housing arrangements and court orders to make them available does not solve the problem as labour remains treated as a low-cost commodity deprived of an agency to bargain for a better deal at work and for proper as well as affordable living space which is denied to them. Circular migration of labour which tears up the social fabric of the working-class household seems to be taken for granted.

The Prayas Centre for Labour Research and Action produced a very timely document on housing for the reserve army of labour adrift in the country and its economy, arguing that these uprooted contingents of the working poor qualify for decent shelter. As real estate politics mainly cater to an upper- to middle-class clientele, the surplus workforce drifting in from the countryside and anxious to settle down in the city finds no access to urban space for low-cost-free housing. Addressing a Joint Session of Parliament on 23 February, 2015, the President of India conveyed the government’s assurance of ‘housing for all’, especially India’s poor (PCLRA 2019). This assurance goes against the manner in which the state-sponsored scheme of infrastructural upscaling with a high-tech bias under the Smart City Mission aims to promote urban modernity and citizen-friendly comfort, excluding the labouring poor who firmly remain deprived of the rights to citizenship. They are deported from the upgraded urban centres and dumped on the city’s outskirts (Breman 2016). The promise of Housing for all cannot be implemented, since both capital and government insist on keeping huge crowds of wage hunters and gatherers without proper shelter rather than end their habitual nomadism: capital, because footlooseness drives down the labour price to the lowest level possible, and the government, because it aims to keep the off-and-on employed men, women and children embedded in the countryside, where it is fragmented and scattered over a multitude of rural slums in self-fabricated tenements, rather than allowing this huge reserve army of labour to settle down *en masse* in poor quality apartment blocks on the urban outskirts (Breman 2016).

## The Flip Side of the Proposal to Register Migrants

The initiative to register migrants who, at present, move around completely unidentified by the local administration is equally spurred on by civil intent of goodwill, i.e. to document the undocumented, and to that extent to decommodify them. Appalled by wanton negligence shown to footloose labour by government agencies and the profound absence of official accountability, ActionAid argues in its latest report that it is vital to establish mechanisms to protect workers and their rights when they migrate for work. These include setting up of migration facilitation centres for maintaining a database of migrant workers at both source and destination districts, providing them information and access to welfare schemes and ensuring their access to grievance redressal processes. The labour codes which are in various stages of being approved or have been promulgated need to be urgently revisited to include provisions for migrant workers. Other protective laws and mechanisms such as local committees for the prevention of sexual harassment at the workplace need to be made accessible to migrant workers (ActionAid 2020). Taken up by the state, the official licensing of these anonymous contingents may very well have the opposite effect. I am therefore quite mistrustful of the National Migrant Information System which is in the offing ‘to monitor and facilitate the smooth movement of migrant workers and their contact tracing during lockdown across the country’ (Times of India 2020). Like the Unique Identification Authority (UID) which raised concerns regarding privacy, security and surveillance, any such proposal must be subjected to intense scrutiny in the manner in which it could seek to regulate the footloose way of life and deficient existence of the migrants. Moreover, it feeds into the build-up of a surveillance state, which is investing larger resources in the Big Brother role of the state rather than health and social security for the labouring poor (Kannan 2020).

How many labour migrants (by end July 2020) retreated to their place of origin in the countryside? Numbers are difficult to come by, but my contention is that the widely varying estimates—from 12 to 30 million—have little plausibility and cannot be relied upon (Wire 2020b). This is because many migrants who had found a niche in the urban economy to ply their trade as street vendors, booth operators and owners of a conveyance (cycle, auto-rickshaw, taxi) or had become skilled as self-employed craftsmen, also joined the flood of casual workers who were leaving for home (Johari 2020). Without earnings, life in the slum they inhabited, with their households, was too costly to sit out the lockdown. Lack of wherewithal forced them to vacate the work and living space they thought to have permanently occupied. The outbreak of the pandemic provided a rare opportunity to close ranks between ‘drifters’ and ‘settlers’ and agitate in public turmoil for an end to containment and cost-free transportation back home. Turning their back on the habitat that wants to get rid of them, feeling cheated and betrayed by both the commanding heights of the state and their employers of the moment, they started to walk out in protest. Certainly, in their cumbersome flight, these hapless flocks strike us as fugitives, but it also shows an agency that has always been denied to them. The labouring migrants not only figure as a staggering multitude in the eyes of beholders, but have also come to realize in their own mind the down and out treatment to which all of them are subjected as subalterns rather than as citizens. Suddenly, these vagrant people seem to have taken the shape of a dangerous class.

There is good reason to suspect that the pandemic mainly affects those who have the least resistance because of the deprivation in which they are accustomed to work and live. Packed together in slums and lacking the hygiene and sanitation required to provide minimum health protection, it is clear that the instructions to exercise social distancing cannot be applied to the densely congested quarters of the labouring poor. Having learnt from bitter experience to endure segregation at periphery of society, they lack the space in their squalid and overcrowded shanties to avoid human contact. It will come as no surprise that the better-off castes and classes see these lowlife quarters as hot spots of the catastrophe that is round the corner. It is clear that the identification of this army of reserve labour as dangerous does not apply to their capability to upset the extremely skewed balance of power.

So what menace does their marginal and often superfluous presence radiate? It is the risk of contagion with the impurity that festers in their ranks and from which the better-off classes must be protected. How and to what effect? This objective is achieved by forcing the fragmented mass into ongoing circulation, chasing them out from their abodes when needed, but also driving them back again to prevent their settlement wherever they go. Scattered over the countryside in communally embedded clusters pre-empts their living jointly in localities which would allow them common space to gather and indulge in concerted action which might destabilize the economic, social and political order that has been established. Home has become a sparse and poverty-stricken waiting room at the village margins or the city’s outskirts, a halt to pass the quarantine that keeps them isolated from the realms of civil society. Generated by today’s political economy, the lockdown on this mass in excess to regular demand was already imposed before the COVID-19 erupted and it is also meant to remain enforced long after the pandemic has worn itself out. To lift the erected barrier of exclusion and insist on their inclusion as a substantial part of the national citizenry in a frame of economic welfare, social emancipation, cultural plurality and political democracy is a forlorn ambit in the given situation of an authoritarian state driven by exceptional ethno-populism and a predatory mode of capitalism.

## Home as a Social Safety Net?

Caught between their places of work and residence, many labour migrants are on both sides of their mobility at risk to progressive marginalization and declining resilience. A large number of households at the bottom of the urban and rural heap are split up in multilocality. This falling apart, of varying frequency and duration, cannot be avoided since those who go off in search of work are not allowed to be accompanied by dependent members of their household too young or too old to work. Not being able to at least reproduce their maintenance cost, they have to be left behind. The impossibility of staying together as a nuclear or joint family continues for many years until health handicaps or physical wear and tear mean that the labour power in the household has been depleted. The idea that only males are prone to depart in search of waged work, as we have said earlier, requires correction. The often-reported finding that females and children add up to at best 10–15% of the footloose workforce ignores their much higher participation in labour nomadism. The ceaseless coming and going of both adults and minors in short or long intervals not only increase the fragility of this primary unit of cohabitation, but also undermine social cohesion and solidarity within it. A difficult to gratify relish for consumerism is driven by surrender to a capitalist mode of conduct that pervades even the milieu of the last and the least. Male providers, in particular, tend to put their own needs before those of dependent members of the family.

In a recent article, Shah and Lerche (2020) have quite rightly pointed out that the research on labour migration in India mainly deals with the predicament of this footloose workforce at the sites of their arrival. Their analysis zooms in on the kinship and family support networks which keep the migrants, even during their absence connected to their home base in a production–social reproduction binary. These informal and also invisible economies of care play a major role in linking those who have departed with the household members left behind, a feature which also needs to be traced in a gendered and age-wise perspective. Their argumentation addresses the relationship between waged work and the informal economies of care and provides important insights into how the appropriation of value from footloose labour is structured. However, I have reservations about their argument of ‘safety nets at home as a fall back option, a support frame of the last resort for the reserve army of labour when in dire need’. Indeed, intra- and extra-household dynamics of care are being reconfigured because of migration, but not necessarily so in a win–win relationship for all stakeholders concerned. The stereotypical opinion is that migrants save and transmit a third to a half of their incomes for the maintenance of dependent members of their households. Yet, I frequently encountered exceptions and other anomalies to this obligation to support close family members on many occasions during my fieldwork. In case of prolonged absence, in particular the bonds keeping the household together often tend to weaken and these long-term migrants also have to face erosion of their social weight in the community on their return. Hybridity at ‘home’ is the price paid for not being there. Sethi (2011) has written a fascinating portrait of such life spent in individualized and stark anonymity. That finding leads me to observe that among the footloose workforce individuation-cum-alienation remains a much underexplored topic of research.

What are the further prospects for the labouring poor who have fled from the mega-cities and rural sites of seasonal or otherwise unsteady and time-bound employment that has turned hostile to them? Back in the village invokes their possible return to agriculture which, until national Independence, used to be the mainstay for the overwhelming majority of the population. The high man to land density already then, the outcome of a colonial policy which, till the end, remained firmly anti-industrial and anti-urban, urged India’s state builders to adopt a strategy which would push the redundant workforce out of their rusticity rather than distributing the appropriated surplus of arable land above the fixed ceiling to land-poor and landless peasants. The dominating castes–classes in the village economy stood to benefit from the land reforms proclaimed in flagrant disregard of the promise made by the Congress government to parcel out the requisitioned property to tillers dispossessed from ownership in the lower rungs of the agrarian ladder. With time, demographic growth has further increased their vulnerability. The small and marginal owners are left with tiny holdings inadequate for viable farming. Consequently, the semi-proletarian and proletarian classes are forced to go off in search of alternative and better livelihood opportunities leaving behind plots which often remain uncultivated. Without property and non-agrarian skills, many of them have failed to establish a permanent foothold away from their place of origin and bring along the dependent members of their household. This is why they invariably have to come back to where they are considered to belong in the nearby or remote hinterland.

In a demographic forecast, a panel of experts have calculated that with a steady slowing down of the growth rate India will overtake China as the world’s most populous country around 2031 to reach 1.52 billion inhabitants by 2036 (National Commission on Population, Population Projections for India and States, 2011–2036). Despite changing demographics and rapidly declining fertility rates, this is deemed to be a worrying prospect. On Independence Day 2019, the Prime Minister expressed his concern over what he called a population explosion. His speech equated acceptance of the small family norm with patriotism and self-reliance. This was followed by spate of draft private member Bills in Parliament incentivizing two child families and disincentivizing larger families, practically tilting towards measures which would be coercive for poor families. Arguably such legislative intent is based on a completely flawed understanding of not only the nature of demographic transition in India, but also the causes of the present economic and employment crisis.

In line with the priority given to boosting the urban economy, it comes as no surprise that much of this decelerated demographic increase will be in towns and cities, which will see a rise in the share of the population from 31% in 2011 (the date of the last census) to 39% in 2036 which implies a decline of the rural population from 69 to 61%. In this projection, internal migration is also factored in, although unrealistically so. To begin with, the assumption is that interstate mobility will continue to be at the same rate as in the past decade. Given the shape of pro-urban and anti-rural politics and policies, the flight from land and village is bound to further increase sharply. Lack of employability and income is as before, the main reason for the exodus of a growing number of people from their rural habitat. But adding to this predicament is my fieldwork-based surmise that for the down and out masses, agriculture and rurality have become scorned as a way of life, an option of the last resort. As has been demonstrated, the urban growth poles make use of the heavily underpaid labour power of the migrants held in reserve, but do not allow them to settle down as co-residents. After a season, a few years or at the end of their working life push back these footloose crowds to where they belong: nowhere. The ability to settle down in urban work and life is a precondition for inclusion into the ranks of citizenship. Many of the labouring poor fall abysmally short of the propensity required—the state of being self-provident above all—to claim this prerogative.

The departure of working members of the resourceless households is above all driven by the imperative need to hunt for a living which is not available locally. However, their homecoming also subordinates them once again to a milieu of steep inequality from which they had hoped to escape. Exploitation and discrimination remain their fate in their employment elsewhere, but in its commodified *avatar* tend to be less personalized and more anonymous than in the village where all co-residents are well aware of their polluted identity as members of scorned communities. That profile does not leave them when adrift, but it tends to be less stultifying and denigrating than in their place of origin. The rule of domination and repression finds expression in the harsh treatment of the labouring poor in the rural landscape inhabited by them and continues to be heavily understated in the classic imagery of consensual village life and lore. In contrast to M.K. Gandhi who praised the organic unity of the late-colonial village and eulogized its communitarian ethos, B.R. Ambedkar, on the basis of his own downgraded identity, portrayed the village as a theatre of darkness in which the rural underclasses were kept stifled in rank subalternity. Brutalized inequality has now become evidently manifest in the treatment meted out by vigilantes from higher up to the subaltern, especially those belonging to the Scheduled Castes, Scheduled Tribes and religious minorities (Breman 2019–2020).

## Back in the Village Facing Immiserization

On their return to the village, this workforce kept footloose may get engaged in agricultural production in the peak periods of the annual cycle, but their full-time involvement in the existent and predatory mode of capitalist tillage is out of the question. What then will give them sufficient respite from hunger? The promised food ration, if fairly and fully distributed, will help to alleviate distress. Public works reluctantly and haphazardly provided under the Mahatma Gandhi National Rural Employment Guarantee Act (MGNREGA) will also give much-needed relief. On 20 June, the Prime Minister announced a new mega scheme, the *Garib Kalyan Rojgar Abhiyan* to employ labour migrants who have gone back to where they came from. The package, launched in pre-election Bihar, is again more cosmetic than heralding a genuine policy shift. It brings no additional resources, but aims to frontload existing ones in 4 months. The programme will concentrate on 116 districts in six states which are officially considered to have seen the return of more than twenty-five thousand migrants each and is meant to upgrade the rural infrastructure. In the words of the Prime Minister: ‘until now you were developing cities… now you will help your village’.

Where I am quite sceptical about the implementation of this intervention is not so much about its limited scope and focus on infrastructural development that is already the prime business of MGNREGA, but in that its objective is at odds with the main objectives of the economic policy axioms foregrounding small government and self-reliance for tackling the dire lack of waged work. The employment provided would not be enough to protect the labouring poor from pauperism. A major finding reported by Mishra et al. (2020) on the impact of the pandemic in their village-based investigations mainly carried out in Odisha between end May and early June 2020 clarifies that cultivation and marketing operations have been severely hampered.

Harvested crops, such as vegetables, could not be sold or were sold at a loss. The closure of weekly markets in the villages and the unavailability of transport to reach ‘mandis’ during the lockdown severely affected perishable crops. Animal husbandry and NTFP collections have been disrupted. Unavailability of fertilisers and pesticides that affected the sowing of new crops was also reported (Mishra et al. 2020: 10).

The data collected indicate that the returned migrants did not come back to a rural economy able and willing to reintegrate them in agricultural production. Summing up the sliding down of the land-poor and landless classes, they conclude that unemployment has much increased in the villages. Livelihoods of the labour households are deeply paralysed. Consumption expenditures have been depressed. Most households are on the verge of spiralling down in debt. I fervently disagree with the current wisdom that the agrarian question has lost its former saliency. While rejected as having become obsolete, cultivable land and how to make use of it should play a crucial role in reflecting on other scenarios than prescribed by political suitability. There is scope for a revitalization of the peasant economy, but preconditional for this to take place is a new round of land reforms restoring the dispossessed classes to their erstwhile property as well as sharing in the restored local commons together with a very fundamental overhaul of agrarian policies and an end to ‘the smart city’ gospel Breman (2019–2020: 212–215). In short, phasing out of neoliberal policies is of imperative urgency.

Civil activism has stepped in where the state not only failed, but also refused to take charge of accelerating misery. The migrant crisis is reported as a health calamity and not as a dismal failure of the government’s pandemic policies (Abhishek 2020). Public opinion has been consistently manipulated by a narrative to which the mainstream press willingly fell prey. The state remains absolved from gross dereliction of duty, although critical reporting on government omissions tends to be avoided in the current style of politics because such negative coverage easily becomes branded as unpatriotic. In a follow-up to Abhishek’s critique, Samaddar (2020) argued that the migrant appeared as a fault line in the nation’s access to essential conditions of life, of health, food and shelter in particular. His comments observed that a form of civil-based solidarity has flared up all over India, prompted by compassion, but also inspired by efforts to change the fabric of society away from majoritarian politics (ibid.). A wide variety of voluntary associations and non-government agencies and activists of acclaimed rights have highlighted the plight of the immiserized workforce unable to cope with the limited means at their disposal.

## A Political Strategy of Cumulative Inequality

What is going to happen once this health crisis has burned itself out? The stalwarts and profiteers in the current regime of exploitation, with the ruling power elite and owners-cum-managers of big capital as its stakeholders, are determined to firmly stay on the course of neoliberalism and resume business as usual. In that eventuality, the workforce made footloose would have no option than to take to the road once again. I find this back to ‘normalcy’ script rather implausible in view of the anxiously awaited recession all around. Over the last half century, the drift to casual or self-employment has worldwide become an organizing principle of the economy, a shift away from formality and cancellation of the standard labour contract for which the lower ranks of the workforce in all continents have paid an extremely high price (Breman et al. 2019). In India, students and activists who are despondently reminiscing about a more congenial landscape of labour in the past should bear in mind that already before the current juggernaut made its impact, Congress had already warmly embraced neoliberalism. Its erstwhile Prime Minister Manmohan Singh insisted that ‘the tyranny of the Inspection Raj must end’ (Sundar 2020; Sarkar 2020). Although steadfastly corrupted when in existence, downgrading this official machinery to zero action also put an end to the pretension of state protection. Investigating the impact of an earlier economic meltdown in 1997–1998 which badly hit the Asian economies and the labouring poor in particular, I summed my findings with the following comment that seems to have lost nothing of its relevance.

The labour market of the informal sector is highly fragmented; those who are laid off in their branch of activity have no alternative but to go back ‘home’, because staying on in the city without earnings is next to impossible. But returning to their place of origin is not a straightforward option, given the lack of space in the rural economy. From the perspective of the world’s underclasses, what looks like a conjunctural crisis is actually a structural one, the absence of regular and decent employment. The massive army of reserve labour at the bottom of the informal economy is entrapped in a permanent state of crisis. The logic suggests a return to nineteenth-century beliefs in the principle and practice of natural inequality. On this view, it is not poverty that needs to be eradicated. The problem is the poor people themselves, who lack the ability to pull themselves up out of their misery. Handicapped by all kinds of defects, they constitute a useless residue and an unnecessary burden. How to get rid of this ballast? (Breman 2009).

India’s record of economic informalization is a special one. Backed up by governance, neoliberal capitalism has seen to it that many people found redundant in the countryside are prevented from settling down where they have gone in search of livelihood. Not allowed to be accompanied by dependents, they have to leave again when there is no further need for their lowly qualified and underpaid labour power. The future’s agenda is anybody’s guess, but it may get even worse for the excluded misfits than it already has been so far. Discussing the plight in which the bottom ranks of footloose labour are entrapped, Shah and Lerche (2020) sum up their ordeal as conditioned by hyper-precarity, a concept coined by Lewis et al. (2015). The term implies more than just vulnerability due to destitution and emerges from the interplay in late capitalist economies between neoliberal markets and highly restrictive labour regimes denying workers (and migrants specifically) freedom of employment. The restrictions imposed, which are backed up by government agencies and exclude access to citizenship and its concomitant welfarism, can close down any alternative to engage in severely exploitative labour. It is an astute appraisal which, however, could have been strengthened by adding a historical perspective. I have no problem accepting the argumentation, referred to by Shah and Lerche for its relevance to labour migration in India, but would like to point out that what is on offer as a contemporary analysis pertinent to globalized capitalism, duplicates what in an earlier *avatar* was addressed as pauperism. Like today’s hyper-precarious poor, the European transformation in the nineteenth century from an agrarian-artisanal to an industrial–urban type of economy-cum-society went accompanied by an exclusionary policy labelling a segment of the working poor as ‘non-deserving’. Habituated to wander around in desperate search of livelihood, this footloose contingent of paupers stood accused of indolence and vagrancy, became resolutely blocked from inclusion in the ranks of citizenship and was firmly kept under state surveillance as a dangerous class. To detach this underclass from mainstream, society was sanctioned by a racialized ideology of exclusion which exposed the downgraded terrain of the nation to internal colonialism. The common ground to the long trajectory of pauperism from past to present is the anti-egalitarian narrative of this ethos and its fierce praxis at the stage of both early and late capitalism (Breman 2016). Shunned and shunted away beyond the gate of civility the predicament of the targeted people is comparable to that of the non-deserving poor subjected to the vicious doctrine of social Darwinism which we had fervently but fancifully hoped never to see back again.
